# Evaluation of genetic effects on the fatty acid composition of intramuscular fat and backfat of the endangered Angler Saddleback pig

**DOI:** 10.5194/aab-69-129-2026

**Published:** 2026-02-20

**Authors:** Anna Olschewsky, Andreas Kleinlein, Dirk Hinrichs, Stefanie Klingel, Georg Thaller, Angela Sünder, Daniel Mörlein

**Affiliations:** 1 Animal Breeding Section, Faculty of Organic Agricultural Sciences, University of Kassel, Nordbahnhofstraße 1a, 37213 Witzenhausen, Germany; 2 Center for Rare and Endangered Domestic Animals, Arche Warder, Langwedeler Weg 11, 24646 Warder, Germany; 3 Institute of Animal Breeding and Husbandry, University of Kiel, Hermann-Rodewald-Straße 6, 24118 Kiel, Germany; 4 Department of Animal Sciences, University of Göttingen, Kellnerweg 6, 37077 Göttingen, Germany

## Abstract

The Angler Saddleback (AS) pig is an endangered breed that originates from Germany. To date there is a lack of scientific research into the meat quality of this local breed, particularly with regard to fatty acid composition. Due to the limited availability of AS piglets, a fattening experiment was conducted with 58 individuals as a pilot study. To meet this slower-growing breed's lower nutritional demands, the pigs were fed a diet rich in silage and low in concentrate feed. In connection with the high backfat (BF) thickness ( M=37.8mm ), increased levels of monounsaturated fatty acids were observed in both the intramuscular fat (IMF; M=46.6% ) and the subcutaneous fat ( M=48.6% ) as compared to other pig breeds. The saturated fatty acids amounts were rather low in intramuscular fat ( M=33.6% ) and in BF ( M=38.7% ), as was to be expected for a fatty pig breed. Furthermore, the proportion of polyunsaturated fatty acids in the BF was very low ( M=12.7% ), despite the high proportion of grass–clover silage in the diet. These results, along with some unexpected correlations among the target variables, might be due to a genetic effect of the AS pig. Therefore, genome-wide association studies (GWASs) were performed using SNP-array data from the 58 pigs. Based on this, 38 significant variants related to pork quality traits were identified. The significant loci were distributed over 10 chromosomes and further underlined the influence of genetics. Given the limited sample size for genomic analysis, these findings can only provide an initial insight. However, the results clearly indicate that examining the fatty acid composition of local pig breeds in more detail could provide valuable information to enhance selection for pork quality.

## 1 Introduction

The Angler Saddleback (AS) pig is a local breed originating from the north of Germany (GEH e.V., 2022). The breed is endangered from extinction given its very low population size of 115 female and 7 male pigs (TGRDEU, 2025). The pig is known for its undemanding (robust) nature in terms of husbandry and feeding, and the meat is appreciated by connoisseurs for its excellent quality (Iversen, 1997). However, the scientific investigation of these positive attributes of the breed had yet to be conducted. Therefore, a fattening experiment was carried out with 58 AS pigs. The sample size was limited because only a few piglets were available due to the small overall population size. As a pilot study, the project aimed to gain initial insights into various characteristics of this endangered breed and assess its conservation value. The first results regarding performance traits were published in a companion article by Olschewsky et al. (2024). As part of the fattening experiment, the present study examined the meat quality of the AS pig breed.

Until the middle of the 20th century, it was common for the AS pig breed to have a high fat content in its meat (Iversen, 1997). This also applies to many other European local pig breeds (Poklukar et al., 2020). However, the demand for low-fat foods has increased in Germany and other western industrial nations since the 1950s (Resurreccion, 2004). With this, the use of fast-growing pigs from crossbreeding programs has become dominant for commercial and large-scale pork production (Knap and Rauw, 2008). In addition, these pig breeds have become more important due to their increased efficiency and productivity. As a consequence, the use of local pig breeds like the AS pig has decreased (Iversen, 1997; Resurreccion, 2004).

Fast-growing pigs provide a high lean meat percentage (LMP), which is associated with a decrease in total fat and an increase in polyunsaturated fatty acid (PUFA) content (Wood et al., 2008; Burnett et al., 2020). PUFAs are known as a healthy component in human diets, especially n-3 fatty acids, with a low ratio of n-6 : n-3 fatty acids being desirable (Dugan et al., 2015). However, due to the lower melting point and a higher oxidation potential, an increase in PUFAs can have negative consequences with regard to sensory and processing traits, e.g., in view of rancidity development (Amaral et al., 2018; Burnett et al., 2020). Contrarily, a higher overall amount of fat is followed by increasing intramuscular fat (IMF) amounts and with this an increase in monounsaturated fatty acids (MUFAs) and saturated fatty acids (SFAs) (Burnett et al., 2020). High amounts of MUFAs and SFAs in pork positively influence both the palatability of fresh meat and the processing properties. The palatability of the meat encompasses key sensory dimensions such as tenderness, juiciness, and flavor (Burnett et al., 2020).

Since the fatty acid composition in pigs is influenced by carcass and meat quality parameters (e.g., proportion of backfat and IMF), a genetic component is to be assumed. In view of this, previous studies reported low to high values for heritability ( $h^{2}=0.004-0.650$ ) for fatty acid composition in different porcine tissues (Zhang et al., 2019a; Catillo et al., 2020; Zappaterra et al., 2020). In addition, feeding is considered to be a key factor influencing the fatty acid composition (Raes et al., 2004). In pigs, which are monogastric animals, dietary fatty acids accumulate in the adipose tissue. It has therefore been shown that diets high in PUFAs (e.g., roughage, linseed oil, or fish oil) can contribute to an increase in this fatty acid fraction in the IMF and backfat (BF) of pigs (Raes et al., 2004; Arkfeld et al., 2015).

In view of the genetic influence on fatty acid composition in pork, causal variants for meat quality and carcass quality traits have been studied to improve selection strategies (Pena et al., 2016). These strategies are mostly intended to minimize negatively correlated carcass and meat quality parameters. For example, causal variants are sought that should make it possible to increase the IMF and thus improve palatability while maintaining a high LMP (Pena et al., 2016; Zhang et al., 2019a). The selection of Duroc pigs is a particular success story at this point. These pigs have a particularly favorable ratio of IMF and LMP (Pena et al., 2016).

To date there is a lack of scientific analyses of meat quality and especially fatty acid composition of the German AS pig and related genetic variants. This breed, however, is particularly interesting as it is known for its rather high carcass fatness, yet it has been fed for decades with less nutrient-dense yet roughage-rich diets (Iversen, 1997). Therefore, the first objective of the present study was to find out how the described selection and feeding conditions have influenced the fatty acid composition of this local pig breed – that is, how genetic factors and feeding with higher PUFA levels influenced the correlations of carcass and meat quality parameters along with fatty acid fractions. The second objective of the study was to identify gene loci that could be associated with the investigated traits of carcass and meat quality.

In summary, the following study aimed at (1) characterizing the fatty acid composition of current-day AS pigs; (2) revealing correlations between carcass quality, meat quality, and fatty acid composition; and (3) detecting associations between the studied traits and genetic loci of AS pigs by means of genome-wide association studies (GWASs).

## 2 Material and methods

### 2.1 Animals and phenotypes

The pigs investigated in this observational study were provided by five breeders from northern Germany. Altogether, 58 purebred animals from herdbook-registered parents were fattened between October 2018 and October 2020 as described in detail by Olschewsky et al. (2024). In two trials, 40 (21 females, 19 castrates) and 18 (10 females, 8 castrates) animals, respectively, were raised at an experimental farm of the University of Kiel under semi-controlled conditions. In the first trial the pigs came from three different breeders, and in the second trial two different breeders delivered the piglets.

The pigs were kept indoors in single compartments with a size of 2 $m^{2}$ per pig. The animals were fed with a commercial diet slightly reduced in energy and protein (Table 1). The daily concentrate amounts were restricted, and, in addition, grass-clover silage was offered daily, i.e., 1.5–3.0 kg fresh matter per pig. This feeding regime was the same for the two trials and was intended to meet the overall lower nutritional demands of this breed and to avoid excessive carcass fat. However, some nutrient fluctuations may have occurred because the concentrate originated from different lots and the silage came from different years of the same farm due to the time interval between the trials.

**Table 1:** Nutrient and energy content of the grower and finisher diets (based on 88 % dry matter) according to feed declaration for two consecutive trials with Angler Saddleback pigs ( n=58 ).

Item	Grower	Finisher
Crude protein (%)	16.0	13.5
Lysine (%)	0.90	0.60
Methionine (%)	0.28	0.18 (trial 1);
		0.19 (trial 2)
Crude fiber (%)	5.3	7.0
Crude fat (%)	3.7 (trial 1);	3.7
	3.6 (trial 2)	
Crude ash (%)	5.4 (trial 1);	5.0
	5.5 (trial 2)	
Ca (%)	0.90	0.65
P (%)	0.55	0.45
Na (%)	0.23	0.20
ME (MJ)	12.6	11.8

Due to the limited availability of the animals, it was not possible to completely synchronize their age. Therefore, the fattening started with a mean age of about 106 d ( SD=21.6 ) in the first trial and about 87 d ( SD=23.7 ) in the second trial. The average initial live weight was 27.5 kg ( SD=7.9 ) in the first trial and 19.2 kg ( SD=8.6 ) in the second. To compensate for the different age of the animals, two slaughter time points were realized for both trials. Furthermore, the fattening period was shorter in the first trial ( M=219d , SD=14.3 ) than in the second trial ( M=235d , SD=20.4 ), resulting in an average age at slaughter of 322 and 325 d. The average live weight at the end of fattening was lower in the first trial ( M=139kg , SD=4.7 ) than in the second trial ( M=152kg , SD=6.0 ) (Olschewsky et al., 2024).

Following the German “Guideline for station testing for fattening performance, carcass value, and meat quality in pigs” (Bundesverband Rind und Schwein e.V., 2019), the post-mortem measurements regarding carcass and meat quality were conducted by trained staff under controlled cooling conditions in a small countryside slaughterhouse. The measurement of LMP was based on the “two-point” method and was calculated using the following formula: LMP=58.10-0.56×backfat measurement (including rind) in mm+0.13×meat measurement (thickness of loin muscle) in mm. BF thickness was calculated as mean values of measurements at three points. Samples for assessing the IMF amount were taken from the loin muscle (*M. longissimus thoracis et lumborum*) between the 13th and 14th rib. With the use of near-infrared spectroscopy (FOSS NIRSystems, Hamburg, Germany), the analysis was performed based on an in-house calibration at the University of Kassel. Further descriptions of the methods are given in Olschewsky et al. (2024). For the analysis of fatty acids, samples were taken from the loin muscle and the subcutaneous fat between the second and third rib 24 h post-mortem. The meat samples were wrapped in plastic bags and transported, under cooled conditions, to the laboratory of the University of Göttingen. There, the samples were vacuum-packed and frozen at -18°C until chemical analysis. For semi-quantitative analysis of fatty acid methyl esters (FAMEs) in BF and IMF, the freeze-dried samples were processed similarly to the method described by Altmann et al. (2020) using gas chromatography coupled with flame ionization detection. The sample preparation according to Altmann et al. (2020) for feed fats was adapted to BF and IMF. As a separation column, a ZB-WAX (30 m, ID 0.32 mm, 0.25 µm) was used, and the analysis conditions were like those of an oven program. A split ratio of the gas chromatograph was modified for the column, and sample material was used. Fatty acids were identified using the Supelco^®^ 37 Component FAME Mix (CRM47885). Some additional standards were used for identification and evaluation purposes (e.g., retention time control) of single fatty acids.

The semi-quantitative determination of the fatty acids was carried out by calculating the proportion of the peak area of the individual fatty acid in relation to the sum of the peak areas of all identified fatty acids. The measurements were performed twice. Afterwards, the overall percentage of SFAs, MUFAs, and PUFAs and the n-3 : n-6 ratios were calculated for the respective individuals.

### 2.2 DNA extraction and genotyping

DNA from 58 AS pigs was isolated from hair follicles, and the “sbeadex™ mini-Kit” (LGC) was used for DNA extraction. Single-nucleotide polymorphism (SNP) genotyping was performed with a semi-customized version of the porcine SNP80K BeadChip (Illumina, San Diego, CA, USA). Due to the overall low population size of the AS pig breed, no animals were excluded based on their relatedness. However, non-autosomal and unmapped SNPs were removed from the dataset. The genomic data were annotated to the *Sscrofa* 11.1 reference assembly and were converted into PLINK (v1.9; Purcell et al., 2007) input files. For the quality control, SNPs that displayed a call rate below 95 % and a minor allele frequency<5% were excluded. No individuals were filtered out due to a low genotype call rate (below 90 %). The final dataset included 58 animals, 42 569 SNPs, and an overall genotyping rate of 0.991.

### 2.3 Genome-wide association studies

GWASs of 58 AS pigs were performed using SNP genotyping data for different carcass and meat quality traits. With the use of Gemma v0.98.5 (Zhou and Stephens, 2012), univariate linear mixed models were analyzed with the following formula:

1 y=Wα+xβ+u+ε,

where y is the vector of phenotypic observations for n individuals. As fixed effects, W is the n×c matrix of covariates where the first column represents the intercept, α is a vector of the corresponding coefficients, and x is the vector of genotypes with β representing the effect size of the markers. Random effects are complemented with $u∼{\text{MVN}}_{n}(0,\lambda r^{-1}K)$ , including the variance of residual errors ( $r^{-1}$ ), the ratio between variance components ( λ ), and the n×n relatedness matrix ( K ), with ${\text{MVN}}_{n}$ being the n -dimensional multivariate normal distribution. With $\varepsilon ∼{\text{MVN}}_{n}(0,r^{-1}I_{n})$ an n -vector of errors was included, with $I_{n}$ denoting an n×n identity matrix.

Significant covariates for each phenotype were derived from the statistical analysis in R (v4.3.3; R Core Team, 2021). Therefore, a principal component analysis (PCA) was performed using the packages “factoMineR” (v2.11) and “factoextra” (v1.0.7) to rule out multicollinearity of possible covariates. The process of standardizing the variables is included by default in the aforementioned packages. Using linear models (lm() function), the significance of the possible covariates “sex”, “breeder”, and “age” was tested afterwards for each target variable. The models included the testing for normal distribution of residuals by plotting the results. Since the residuals of all examined phenotypes were normally distributed, no further transformation of the phenotypic data was necessary. In addition, a standardized relatedness matrix was calculated using the genomic information of 58 AS pigs with the related option in GEMMA. Both the covariates and the relatedness matrix were included in the GWASs.

Using the Wald statistic, the association of each SNP was tested. The Bonferroni correction for multiple testing was implemented with the significance level set to $p\leq 1.2\times 10^{-6}(0.05/\text{number of analyzed SNPs})$ . For the comparison of the results of the AS pig with previous studies, the significant variants were mapped against the *Sscrofa* 10.2 reference assembly afterwards. For the identification of candidate genes, a region of 100 kb on either side of the position of the significant variants was set. With the use of biomaRt (v2.58.2; Durinck et al., 2009), the “sscrofa_gene_ensembl” dataset was explored to identify genes within the defined windows.

### 2.4 Statistical analysis

Further statistical analyses were conducted using R. With the use of the packages “ggplot2” (v3.5.0), “ggdist” (v3.3.2), and “ggthemes” (v5.1.0), the differences in the fatty acid classes between the two trials were visualized. With the core.test() function, pairwise correlations between fatty acid classes and carcass, along with meat quality parameters, were computed. In the case of a normal distribution of both variables, the “Pearson” method was used. If at least one variable deviated from a normal distribution, the “Spearman” method was applied. For the visualization of the connections between selected target variables of fatty acid composition and carcass, along with meat quality, a PCA was displayed using again the packages “factoMineR” and “factoextra”. All calculations relating to the fatty acid composition, carcass, and meat quality traits were performed independently of the pigs' sex.

### 2.5 Declaration of generative AI and AI-assisted technologies

During the preparation of this work, the authors used DeepL Write in order to improve the language. After using this tool, the authors reviewed and edited the content as needed and take full responsibility for the content of the publication.

## 3 Results

### 3.1 Fatty acid composition of IMF and BF

On average, an IMF amount of M=2.6% ( SD=0.8 , CV=30.4 ) was reached. The fatty acid composition of IMF in the loin muscle was dominated by MUFAs ( M=47% ). Oleic acid (C18 : 1n9c) represents by far the most abundant single fatty acid within this fraction (Table 2). SFAs formed the second-largest class of intramuscular fatty acids ( M=34% ), with palmitic acid (C16 : 0; M=21% ) followed by stearic acid (C18 : 0; M=11% ) having the highest proportions. Overall, PUFAs had the lowest proportion in the intramuscular fatty acid composition ( M=20% ) dominated by linoleic acid (C18 : 2n6c). The n-6 : n-3 ratio is averaged at 13, as shown in Table 2.

**Table 2:** Selection of fatty acids (% of total fatty acids) in intramuscular fat (IMF) and backfat as mean values of two consecutive trials with female and castrated Angler Saddleback pigs (n=58) .

	IMF		Backfat	
	Mean	SD	Mean	SD
Total SFA, %	33.6	2.1	38.7	2.4
C14 : 0	0.9	0.2	1.0	0.1
C16 : 0	21.2	1.6	23.3	1.2
C18 : 0	10.8	1.1	13.8	1.4
Total MUFA, %	46.6	5.9	48.6	1.7
C16 : 1	2.6	0.4	1.6	0.3
C18 : 1n9c	40.2	4.6	44.0	1.6
C18 : 1n9t	2.9	2.0	1.7	1.1
Total PUFA, %	19.8	7.4	12.7	1.7
Total n-6 PUFA, %	18.0	7.3	10.5	1.4
C18 : 2n6c	14.0	5.0	10.3	1.4
C18 : 3n6	0.2	0.2	0.0	0.0
C20 : 3n6	0.4	0.2	0.1	0.0
C20 : 4n6	3.3	2.0	0.1	0.0
Total n-3 PUFA, %	1.5	0.5	1.7	0.5
C18 : 3n3	0.9	0.3	1.3	0.5
C20 : 3n3	0.1	0.0	0.3	0.1
C20 : 5n3	0.4	0.2	0.0	0.0
C22 : 6n3	0.1	0.1	0.0	0.1
n-6 : n-3 ratio	12.8	7.0	7.1	2.6

MUFA, monounsaturated fatty acid; PUFA, polyunsaturated fatty acid; SD, standard deviation; SFA, saturated fatty acid.

For BF thickness M=37.8mm ( SD=6.1 , CV=16.2 ) was assessed, whereas LMP reached M=47.0% ( SD=4.8 , CV=10.1 ). With a slightly higher mean of M=49% than reported for IMF, MUFA had also the highest share in the composition of BF in the analyzed pork. Oleic acid averaged at 44 % and dominated therefore this class of fatty acids. The average amount of SFA ( M=38.7% ) in BF was distinctly higher than in IMF, while palmitic (23 %) and stearic acid (14 %) had the highest proportions.

With a mean of 13 %, the proportion of PUFAs was lower than in IMF. This class was again dominated by linoleic acid (10 %) but by a slightly higher share of n-3 fatty acids (Table 2).

**Figure 1:**
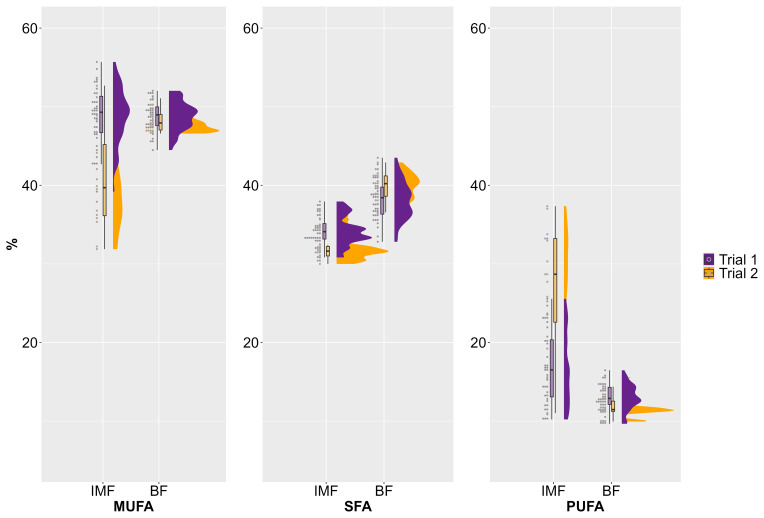
Variation of fatty acid composition (%) in intramuscular fat (IMF) and backfat (BF) between and within two trials with female and castrated Angler Saddleback pigs ( n=58 ), Abbreviations: MUFA, monounsaturated fatty acid; PUFA, polyunsaturated fatty acid; SFA, saturated fatty acid.

Some differences in fatty acid composition can be observed between the two trials, particularly in the IMF of the assessed AS pigs (Fig. 1). The MUFA content in IMF was higher in the first trial ( M=49% ) than in the second trial ( M=41% ). Here, a large variation between the individual animals is noticeable. However, hardly any differences in the MUFA content in BF were found between the trials, and the variation between the individual animals was clearly lower (Fig. 1).

For SFAs, less distinct differences between the trials were detected for both IMF and BF (Fig. 1). In contrast, clear differences between the two trials can be seen for the proportion of PUFAs in IMF. While the average for the first trial was 17 %, it was 27 % in the second trial. Furthermore, a particularly large variation between individual animals can be observed in both trials for the amount of PUFAs in IMF. In contrast, there were almost no differences in the PUFA content of the BF between the two trials, and the variation was clearly lower (Fig. 1).

The n-6 : n-3 ratio in IMF underlines with M=9 (first trial) and M=23 (second trial) that not only the overall proportion but also the composition of PUFAs were clearly different between the two trials. This was overall reflected by a higher proportion of n-6 fatty acids in the second trial. Although there are almost no differences between the two trials for the PUFA content of BF (13 %, first trial vs. 12 %, second trial), the n-6 : n-3 ratio shows a deviation with 5 (first trial) and 11 (second trial). This is due to a decreased amount of n-3 PUFAs in the first trial as compared to the second trial while the proportion of n-6 PUFAs remained largely the same.

### 3.2 Correlations of fatty acid composition with carcass and meat quality parameters

**Figure 2:**
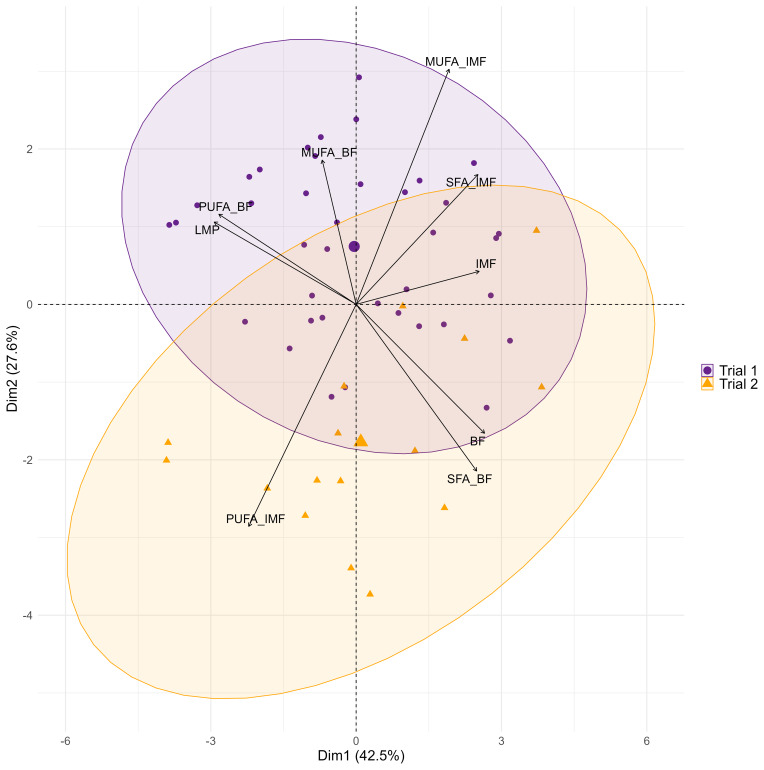
Bi-plot of principal component analysis of selected target variables of fatty acid composition, carcass, and meat quality in two consecutive trials with Angler Saddleback pigs ( n=58 ). Abbreviations: BF, backfat; IMF, intramuscular fat; LMP, lean meat percentage; MUFA, monounsaturated fatty acid; PUFA, polyunsaturated fatty acid; SFA, saturated fatty acid.

A multivariate analysis of carcass, meat quality, and fatty acid traits (Fig. 2) shows that SFAs and MUFAs in IMF are positively correlated with the amount of IMF while being negatively correlated with the amount of PUFAs in IMF.

However, MUFAs and SFAs in IMF appear to be independent of the BF ( r=0.03-0.17 , Fig. 1). In addition, only minor correlations were found between the fatty acid composition of IMF and the amount of LMP ( r=-0.24-0.30 ). A higher BF thickness is associated with a higher SFA in BF, while the amount of PUFAs is rather negatively correlated. Accordingly, PUFA content in BF increases with LMP. A negative correlation is found between SFAs and MUFAs ( r=-0.71 ) in BF. Overall, this multivariate analysis suggests a rather low dependence of fatty acid composition in IMF and BF of AS pigs ( r=0.21-0.34 ).

### 3.3 Genome-wide association studies

**Figure 3:**
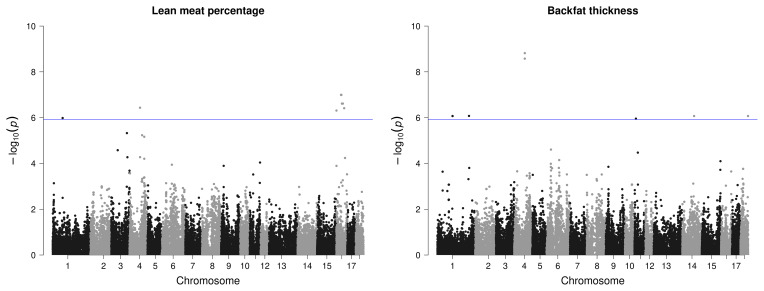
Manhattan plots of genome-wide association studies for carcass quality parameters in two trials with female and castrated Angler Saddleback pigs ( n=58 ).

**Figure 4:**
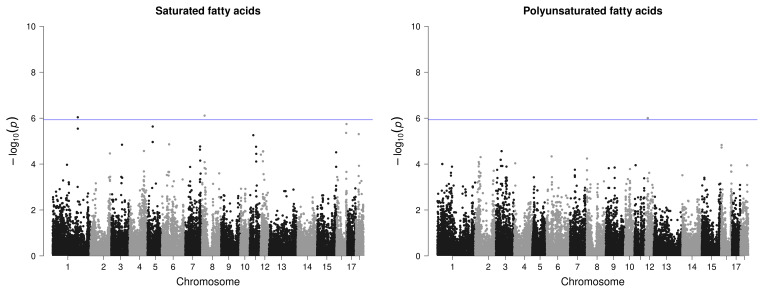
Manhattan plots of genome-wide association studies for fatty acid composition in intramuscular fat in two trials with female and castrated Angler Saddleback pigs ( n=58 ).

**Figure 5:**
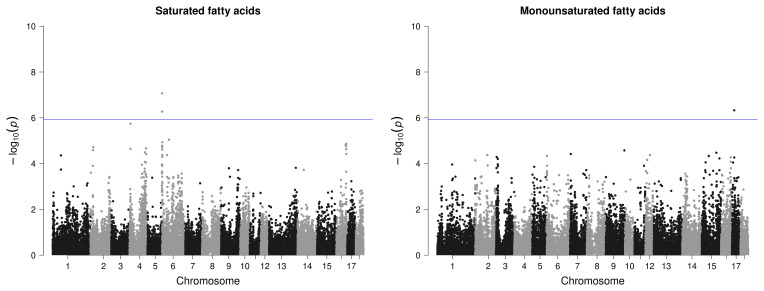
Manhattan plots of genome-wide association studies for fatty acid composition in backfat in two trials with female and castrated Angler Saddleback pigs ( n=58 ).

In total, 38 significant variants located on 10 chromosomes were identified in the GWASs as being associated with the targeted carcass quality, meat quality, and fat quality traits of the 58 AS pigs (Table 3 and Figs. 3–5).

**Table 3:** Significant variants identified by genome-wide association studies (GWASs) for carcass traits, meat quality traits, and fatty acid composition traits in intramuscular fat and backfat in two trials with female and castrated Angler Saddleback pigs (n=58) .

Trait	Chr	SNP	Position (bp)	p value	Candidate genes
LMP	1	ALGA0004254	71 879 005	$1.04\times 10^{-6}$	
LMP	1	MARC0090233	71 930 139	$1.04\times 10^{-6}$	
BF	1	WU_10.2_1_121081951	109 699 420	$8.51\times 10^{-7}$	
BF	1	WU_10.2_1_121150954	109 799 712	$8.51\times 10^{-7}$	
SFA IMF	1	ASGA0005399	182 153 319	$9.19\times 10^{-1}$	TXNDC16, GPR137C
BF	1	ASGA0005967	229 322 493	$8.41\times 10^{-7}$	PCSK5, RFK
LMP + BF	4	M1GA0005986	76 734 449	$3.68\times 10^{-7}$	RP1, SOX17
BF	4	ASGA0020384	76 793 946	$1.52\times 10^{-9}$	
SFA BF	6	WU_10.2_6_2308911	2 207 588	$5.34\times 10^{-7}$	
SFA BF	6	WU_10.2_6_2303592	2 212 895	$5.34\times 10^{-7}$	
SFA BF	6	WU_10.2_6_2637325	2 430 990	$5.34\times 10^{-7}$	
SFA BF	6	WU_10.2_6_2658667	2 452 327	$8.49\times 10^{-8}$	FOXL1
SFA IMF	8	ALGA0046782	18 552 408	$7.79\times 10^{-1}$	DHX15
BF	11	H3GA0031288	10 242 876	$1.10\times 10^{-6}$	RFC3
PUFA IMF	12	WU_10.2_12_16975146	16 950 392	$9.99\times 10^{-7}$	CDC27, KANSL1
PUFA IMF	12	ALGA0122190	17 991 464	$9.99\times 10^{-7}$	PLEKHM1
PUFA IMF	12	ASGA0092611	17 991 464	$9.99\times 10^{-7}$	ARHGAP27
BF	14	ASGA0064884	85 375 815	$8.51\times 10^{-7}$	LRIT2, LRIT1, GHITM, GPR15LG, CDHR1
LMP	16	WU_10.2_16_233939	510 574	$4.90\times 10^{-7}$	CTNND2
LMP	16	ASGA0073029	33 947 925	$1.01\times 10^{-7}$	
LMP	16	ASGA0073034	33 990 830	$1.01\times 10^{-7}$	
LMP	16	ASGA0073036	34 002 361	$1.01\times 10^{-7}$	GZMA, CDC20B, GPX8, MCIDAS, CCNO, DHX29,
					MTREX, PLPP1, SLC38A9, DDX4
LMP	16	H3GA0046467	34 379 749	$1.01\times 10^{-7}$	
LMP	16	WU_10.2_16_36653844	34 545 055	$1.01\times 10^{-7}$	
LMP	16	ASGA0089842	34 598 827	$1.01\times 10^{-7}$	
LMP	16	MARC0027943	34 817 847	$1.01\times 10^{-7}$	
LMP	16	ALGA0090285	35 059 093	$1.01\times 10^{-7}$	IL31RA, IL6ST, MAP3K1
LMP	16	ALGA0090287	35 071 496	$1.01\times 10^{-7}$	
LMP	16	MARC0074483	35 444 990	$1.01\times 10^{-7}$	
LMP	16	ALGA0090328	35 739 611	$1.01\times 10^{-7}$	
LMP	16	ASGA0073088	35 775 489	$1.01\times 10^{-7}$	
LMP	16	ASGA0073091	35 834 227	$1.01\times 10^{-7}$	
LMP	16	INRA0051543	36 302 651	$1.01\times 10^{-7}$	GPBP1
LMP	16	ASGA0073200	40 451 428	$2.43\times 10^{-7}$	
LMP	16	ALGA0090654	46 994 968	$2.43\times 10^{-7}$	
LMP	16	WU_10.2_16_61159651	56 556 518	$3.87\times 10^{-7}$	TENM2
MUFA BF	17	INRA0052828	16 461 796	$4.71\times 10^{-7}$	
BF	18	CASI0001308	55 219 733	$8.51\times 10^{-7}$	POU6F2, VPS41

BF, backfat; Chr, chromosome; IMF, intramuscular fat; LMP, lean meat percentage; MUFA, monounsaturated fatty acid; PUFA, polyunsaturated fatty acid; SFA, saturated fatty acid.

Most SNPs that passed the significance threshold were associated with LMP; they were mainly located on SSC16 with a QTL region between 33.9–56.5 bp. This region was found to harbor 15 different candidate genes (Table 3). Seven significant SNPs distributed over SSC1, SSC4, SSC14, and SSC18 were found to be related with the BF thickness. In view of this trait, the region on SSC4 with two variants stands out due to its comparatively high significance (Fig. 3) and two identified candidate genes.

However, for IMF, no SNP passed the defined threshold, nor could associations be detected for the amount of MUFAs in IMF or for the proportion of PUFAs in BF. Only one variant with rather low significance was found for the proportion of SFAs in the IMF, however, with two candidate genes in the surrounding area. For PUFAs in IMF, a region on SSC12 with three significant SNPs and four candidate genes was identified (Fig. 4).

For the proportion of MUFAs in BF, only one SNP passed the threshold on SSC17, which is clearly distinguishable from the other variants (Fig. 5). However, no candidate gene was found here. For SFA in BF, the region on SSC6 (2.2–2.4 bp) was identified as relevant with four significant variants (Fig. 5) but only with one potential candidate gene.

## 4 Discussion

### 4.1 Fatty acid composition of IMF and BF

Overall, the results underline that the AS pig can still be considered a fatty pig breed, especially with regard to its carcass fatness, which herein was assessed using BF thickness, and its correspondingly lower estimated LMP as compared to commercial pig breeds in Germany (LfL, 2025). In addition, its IMF ( M=2.6% ) is higher as compared to commercial pig breeds (LfL, 2025). However, the IMF of the AS breed is only slightly above the 2.5 % threshold where a positive effect on palatability can be assumed (Poklukar et al., 2020). Only marginally higher results are reported for Krškopolje pigs from Slovenia with IMF=3% (Tomažin et al., 2018), whereas for other local pig breeds, significantly higher values for IMF have been found, e.g., for the Alentejano ( IMF=6.7% ) and Bísaro ( IMF=5.5% ) breeds from Portugal (Martins et al., 2020) and for the Moravka breed from Serbia ( IMF=7% ; Savić et al., 2017). Distinctly higher IMF amounts were also reported for the closely related German Saddleback pig ( IMF=5.2% ; Nürnberg et al., 2013).

In the present study, the MUFA level in IMF of AS pigs ( M=47% ) was rather at the lower end of the range of MUFA=46%-58% reported by Poklukar et al. (2020) for different local pig breeds and for the closely related German Saddleback pig (Nürnberg et al., 2013). In BF, MUFA levels of AS pigs ( M=49% ) were in line with findings in various local breeds (Poklukar et al., 2020), while considerably lower MUFA levels ranging from 34 % to 44 % were also previously found for local breeds (Nürnberg et al., 2013; Crespo-Piazuelo et al., 2020; Chernukha et al., 2023). Therefore, MUFA levels observed for AS appear to be comparatively high. As reviewed by Burnett et al. (2020), higher MUFA contents were to be expected because of the high BF thickness. With oleic acid (C18 : 1) representing the most abundant single fatty acid within this group, this might be, according to the authors, proof of the praised palatability of this breed. However, the amount of SFAs in IMF ( M=34% ) was at the lower end of the range reported for various local and commercial breeds (34 %–44 %) (Nürnberg et al., 2013; Poklukar et al., 2020; Arjin et al., 2021; Grela et al., 2021). In BF, the average SFA level of AS pigs ( M=39% ) was within the range reported for various commercial and local breeds (Nürnberg et al., 2013; Poklukar et al., 2020; Quander-Stoll et al., 2021; Chernukha et al., 2023). The AS meat therefore appears to have a rather low proportion of SFAs in IMF and, in the case of the BF, no distinctly increased level as compared to other pig types. The rather unexpectedly low levels of SFAs could be linked to the feeding of the pigs with the daily offered silage and therefore a diet high in crude fiber and potentially also high in PUFAs. This could also be the reason why the proportion of PUFAs in IMF was clearly higher than the results reported for commercial and local breeds (Nürnberg et al., 2013; Viterbo et al., 2018; Huang et al., 2020; Grela et al., 2021). In contrast to that, the amount of PUFAs in the BF of AS pigs was distinctly lower than reported for various commercial and local breeds (Crespo-Piazuelo et al., 2020; Arjin et al., 2021; Quander-Stoll et al., 2021). Higher PUFA amounts were also reached for the closely related German Saddleback pig (Nürnberg et al., 2013). This contradicts the findings of Quander-Stoll et al. (2021), who investigated groups of a commercial pig breed fed with conventional and organic diets, respectively. The latter was characterized by higher amounts of unsaturated fatty acids and resulted in a significantly increased amount of PUFAs in BF ( M=16% ) compared to the results for AS pigs ( M=13% ).

A noticeable variation in performance and carcass traits of the AS pigs between and also within the animals of different breeders was described by Olschewsky et al. (2024). This is also reflected herein in rather high standard deviations of fatty acid composition traits caused by a variation between the two trials and by animal-specific differences within the trials. Remarkably, the fatty acid composition varies more in IMF than in BF. In view of this, clear differences in MUFAs and PUFAs in IMF were found between the two trials. These unexpected results suggest that there must have been differences in the composition of the feedstuffs that could explain the differences in fatty acid composition between the trials. The feeding regimens in the two trials were the same. Possible nutrient fluctuations, however, could be explained by the concentrate coming from two lots and the silage coming from the same farm but from different years. Unfortunately, no feed samples were available for chemical analysis for nutrient composition or FAME. It has to be underlined that the absence of this information limits the study's findings. However, genetic differences between individual animals, independent of feeding, could also be responsible for the variation. In fact, the animals were provided by five different breeders. Therefore, their different genetic backgrounds could also be responsible for the variation in fatty acid composition of IMF between the two trials. Interestingly, the fatty acid composition in the BF did not differ between the two trials except for a slightly reduced proportion of n-3 PUFAs in the second trial. There has been also less variation between individuals. Although the variation in the fatty acid composition of the IMF indicates an influence, the composition of the BF in AS pigs appears to be less sensitive to (assumed) feed differences and possibly with less genetic variation between individuals from different breeders. This may reflect decades of selection for high fat content and high-quality BF in this breed. This selection took place under heterogeneous feeding conditions with mainly regional feedstuffs. These key breeding objectives were pursued until the middle of the 20th century, and the feeding conditions are still practiced today (Iversen, 1997). These developments could explain why BF traits show less variation, while all other economically relevant traits (e.g., IMF, LMP, daily gain) are highly variable between individuals.

### 4.2 Correlations of fatty acid composition with carcass and meat quality parameters

Some expected correlations can be derived from the fatty acid composition of IMF and BF in the investigated AS pigs with regard to carcass and meat quality traits. As regards IMF composition, a higher amount of IMF is associated with higher proportions of SFAs and MUFAs but lower shares of PUFAs. For BF, a positive correlation of SFAs with carcass fatness is found, which in turn is negatively correlated with the PUFA content and the total amount of LMP. These correlations have been described in the review of Burnett et al. (2020). This is mainly based on the relationship that as pork fatness increases, the proportion of SFAs and MUFAs generally becomes higher, while the proportion of PUFAs decreases (Yang et al., 2010; Burnett et al., 2020). It is therefore rather unexpected that MUFAs and SFAs in IMF in AS pigs are independent of carcass fatness. While this is partly consistent with Yang et al. (2010), who found only small correlations ( r=0.33 ) between IMF and other fat depots, it does not explain why the MUFA content in BF in AS pigs is independent of carcass fatness, which is an unexpected correlation. It is also unexpected that the fatty acid composition in the IMF is less dependent on the LMP, whereas Burnett et al. (2020) state that an increase in the amount of PUFAs is accompanied by an increase in the LMP content. In summary, the analyses for the AS pig indicate that the fatty acid composition in the IMF and in the BF are characterized by only small positive correlations. If diet is considered to be the main factor influencing fatty acid composition in pigs (Burnett et al., 2020), a high correlation can be expected. With this in mind, Yang et al. (2010) found consistently positive correlations between fatty acid fractions in abdominal subcutaneous adipose tissue and lean meat ( r=0.4-0.5 ) in their study of crosses between commercial and local pig breeds. However, the authors point out the lack of high correlations ( r>0.6 ) and conclude that there must also be a genetic influence. This is supported by the fact that low to high heritabilities have been found for fatty acid composition in different tissues of pork (Catillo et al., 2020; Zappaterra et al., 2020; Zhang et al., 2019a). Therefore, a number of authors conclude that selection for a favorable fatty acid composition (e.g., with respect to nutritional value for humans) in pork seems to be possible, to some extent independent of carcass fatness and IMF (Yang et al., 2010; Pena et al., 2016; Zhang et al., 2019a). It should be pointed out that the AS pigs were comparatively old at slaughter compared to common practice. The age of pigs is also known to influence carcass quality parameters, for example, the proportion of subcutaneous fat increases over time (Wood et al., 2008). This could also have contributed to unexpected results and leads to a reduced comparability of the results of the AS pigs.

### 4.3 Genome-wide association studies

The descriptive analysis of carcass, meat, and fat quality traits and their correlations in AS pigs suggests that genetic effects may be an influencing factor in addition to feeding. Therefore, the search for associated QTL regions and potential candidate genes seems relevant. The most obvious region was identified on SSC16, which is related to the proportion of LMP. There were 21 significant variants that passed the significance threshold and were associated with 15 candidate genes. Crespo-Piazuelo et al. (2020) found an association of the same region in crossbred pigs based on the Iberian pig breed but with a connection to the amount of MUFAs and SFAs in BF. In line with this, Amaral et al. (2019) identified the same region on SSC16 being connected to SFAs in Iberian pigs. This region was also identified as being associated with SFA and MUFA content in commercial pig breeds (Zhang et al., 2016, 2019b) and in Chinese Laiwu pigs with regard to SFAs in IMF (Zhang et al., 2019b).

The regions associated with the BF thickness are spread across various chromosomes, with particular significance and two candidate genes on SSC4. However, a significant locus for traits of meat quality was detected neither in the named region on SSC4 nor on SSC1 and SSC18 in previous studies. In contrast, the regions identified on SSC11 and SSC14 with six candidate genes have been described in previous studies. For example, Muñoz et al. (2013) also found a genetic association with SFAs in BF on SSC11 and SSC14 in crosses based on the Iberian pig.

The identified regions that are associated with the SFAs in the IMF of the AS pigs are less distinct from other gene loci in view of their significance, despite harboring three candidate genes. These regions have also not been mentioned in previous studies. Three significant SNPs on SSC12 could be associated with the proportion of PUFAs in the IMF of AS pigs, and they harbor four candidate genes. This region was also identified by Muñoz et al. (2013) to be associated to fatty acid composition, yet that study rather found a connection with SFAs in BF. Another key region of the AS pig is located on SSC6 and is linked to the proportion of SFAs in BF with a single candidate gene. However, this region was not identified as relevant in previous studies. For MUFAs in BF of AS pigs, only one SNP passed the threshold, albeit with a clear distance to other variants. Although no candidate genes are found here, this region has also been identified by Zappaterra et al. (2018) as being associated to PUFAs in BF in Italian Large White pigs.

In summary, some of the loci identified as being related to carcass and meat quality traits in AS pigs have already been described as relevant in previous studies. In contrast, there are a number of GWASs on carcass, meat, and fat quality traits whose significant gene loci do not match the results for the AS pig (e.g., Muñoz et al., 2018; Criado-Mesas et al., 2019; Pena et al., 2019; Óvilo et al., 2022; Liu et al., 2024). Furthermore, a number of candidate genes have been described in detail, such as ELOVL6 (SSC8) (Corominas et al., 2013), ELOVL7 (SSC16) (Yang et al., 2013; Zhang et al., 2017), IGF2 (SSC2) (Criado-Mesas et al., 2019), and SCD (SSC14), as well as LEPR (SSC6) (Ros-Freixedes et al., 2016), which were not detected within the defined scope of the search in the AS pig. This could be because the relevant mutations are not present in the AS pig, as there has been less selection for meat quality parameters, particularly in the recent past. This is consistent with the findings of Muñoz et al. (2018), who identified very different degrees of segregation for relevant alleles within candidate genes for meat quality traits in different local pig breeds. The authors pointed out the polygenic nature of fatness and meat quality traits.

The small sample size for the GWASs performed in AS pigs may have reduced the quality of the analysis (Ziyatdinov et al., 2021; Politi et al., 2023). Nevertheless, given the low population size of the AS being an endangered breed, only this limited sample size could be achieved, and no further reduction of relatedness was possible. Therefore, the results of the GWASs are considered to be the first insights into the genetic architecture of this breed. The characteristic fatty acid composition seems to be noteworthy. It indicates the possible importance of local pigs like the AS breed, which may have extraordinary genetic diversity and uniqueness in terms of carcass, meat, and fat quality traits. To gain further insight, a fine mapping of the identified gene loci and the significance of the identified candidate genes is needed.

## 5 Conclusions

The present pilot study provides, for the first time, a detailed insight into the carcass and meat quality of the endangered AS pig breed. Considering the fatty acid composition, greater variability was shown in the IMF than in the BF. This indicates the influence of feeding on the one hand and genetic effects on the other. The latter is emphasized by the presence of unexpected correlations between carcass, meat, and fat quality traits. These include, in particular, the independence of the fatty acid composition in IMF from overall carcass fatness and from the fatty acid composition in BF. These unique phenotypic traits support the existing conclusion that there are further opportunities to optimize meat quality in terms of palatability, human nutritional value, and feed sustainability. Therefore, the results underline the importance of the potential contribution of the AS pig breed to the optimization of selection for meat quality. Particular attention should be paid to the identified loci that have not been previously identified as significant. Ultimately, endangered breeds such as the AS pig may provide a unique genetic diversity in terms of fatty acid composition and may therefore also serve as a model for further breeding for meat quality in commercial breeds. The results therefore underscore the importance of conserving the AS pig breed.

## Data Availability

The data on fatty acid composition, carcass traits, and meat quality traits are openly available: 10.48662/daks-29.2 (Olschewsky et al., 2025). The genomic data can be requested from the authors.
